# Supplementary material for: A one-gate elevator mechanism for the human neutral amino acid transporter ASCT2
Source: Nat Commun. 2019 Jul 31;10:3427. doi: 10.1038/s41467-019-11363-x (PMC6668440; doi:10.1038/s41467-019-11363-x)
Supplement: Supplementary file 1 — Supplementary Information [file 41467_2019_11363_MOESM1_ESM.docx]

**Supplementary Information for:**

A one-gate elevator mechanism for the human neutral amino acid transporter ASCT2

A. A. Garaeva et al.


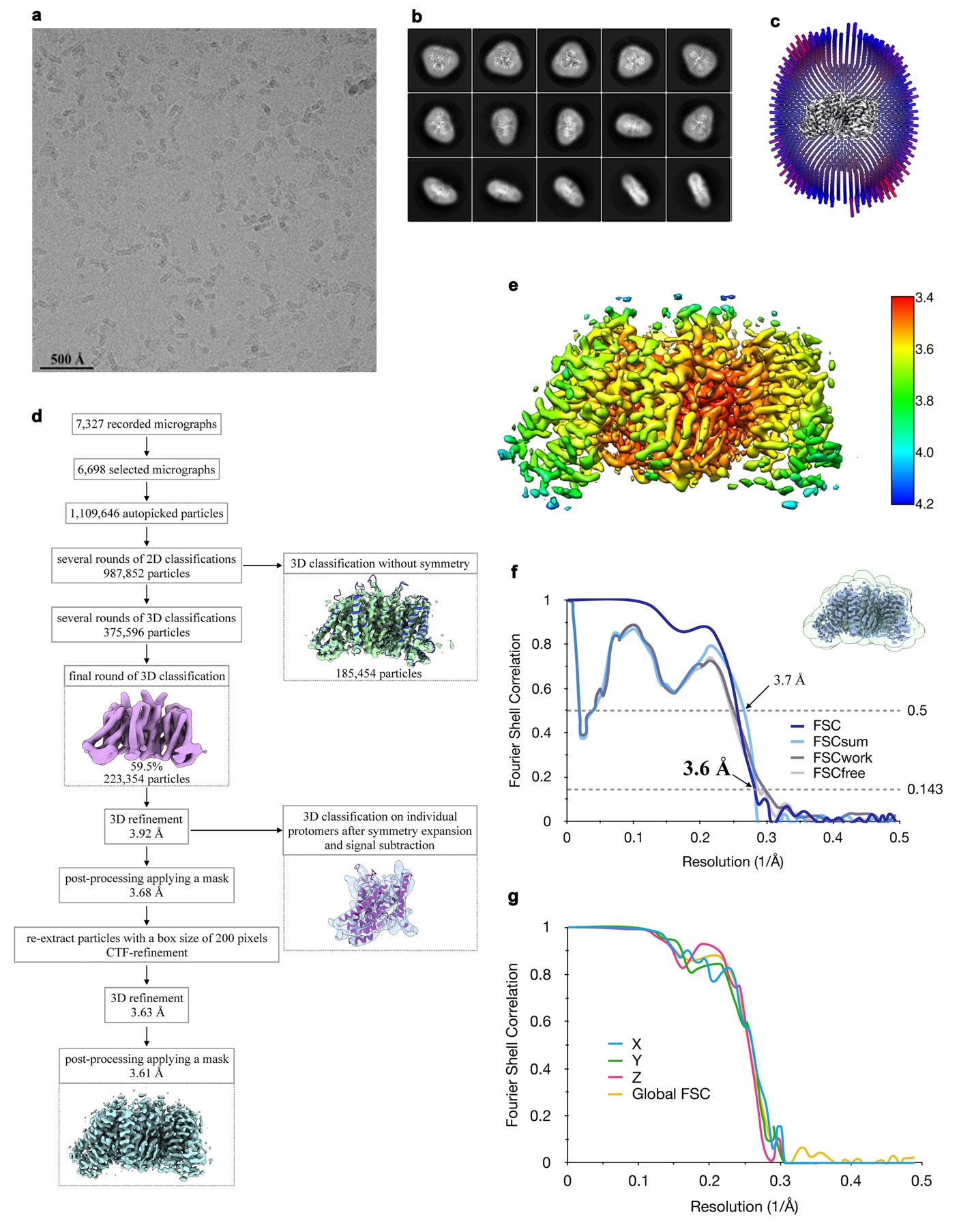


**Supplementary Figure 1. Cryo-EM reconstruction of ASCT2_C467R_-TBOA.** Representative cryo-EM image (**a**) and 2D-class averages (**b**) of vitrified ASCT2_C467R_ in presence of TBOA. **c**, Angular distribution plot of particles included in the final C3-symmetrized 3D reconstruction. **d**, Image processing work flow. **e**, Final reconstructed map coloured by local resolution, as estimated in Relion. **f**, FSC plot used for resolution estimation and model validation. The gold-standard FSC plot between two separately refined half-maps is shown in blue and indicates a final resolution of 3.6 Å. The FSC model validation curves for FSCsum, FSCwork and FSCfree, as described in material and methods, are shown in light blue, dark grey and light grey respectively. A thumbnail of the mask used for FSC calculation overlaid on the map is shown in the upper right corner. Dashed lines indicate the FSC thresholds used for FSC of 0.143 and for FSCsum of 0.5. **g**, Anisotropy estimation plot of the final map. The global FSC curve is represented in yellow. The directional FSCs along the x, y and z axis are displayed in blue, green and red, respectively.


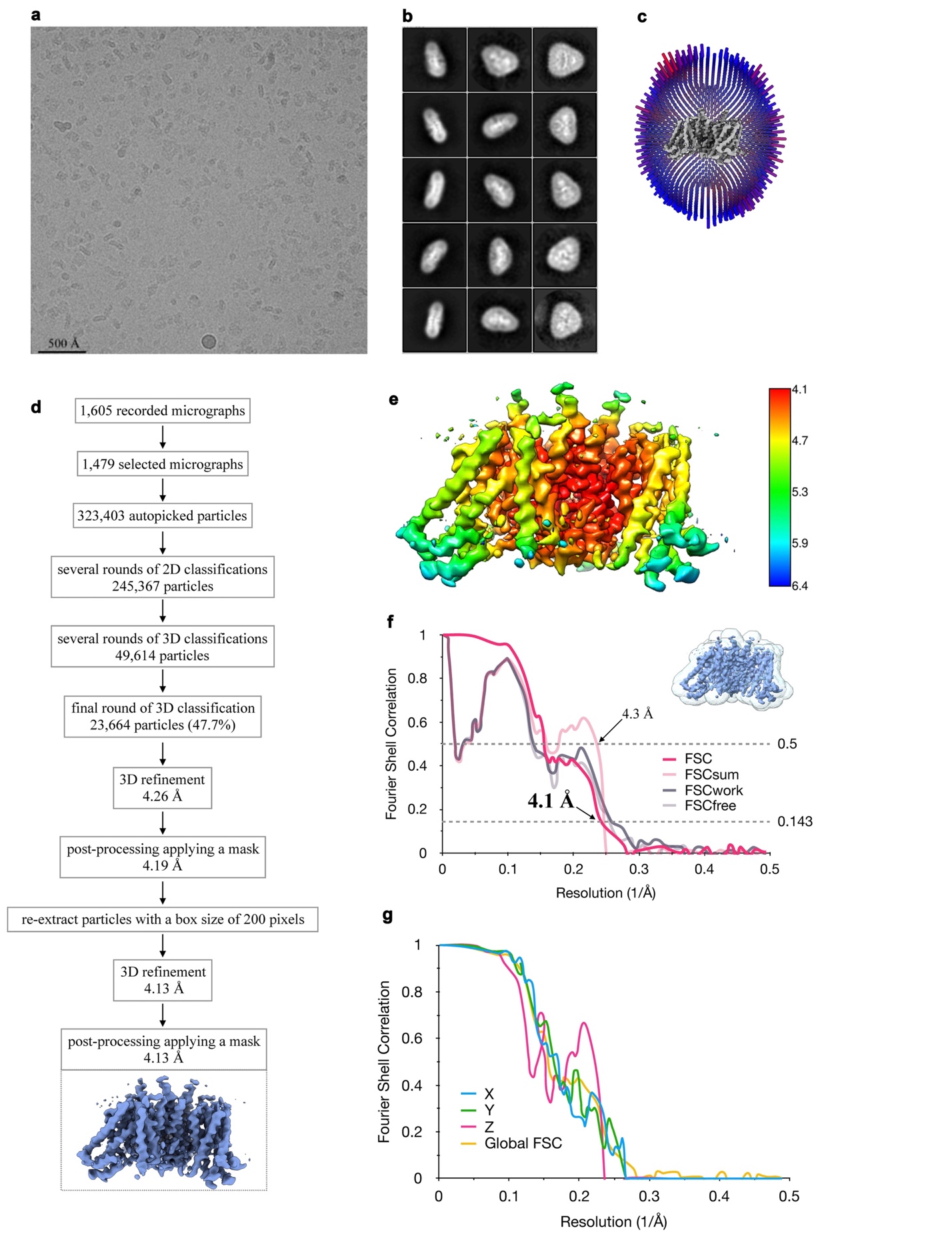


**Supplementary Figure 2: Cryo-EM reconstruction of substrate-free ASCT2_C467R_**. Representative cryo-EM image (**a**) and 2D-class averages (**b**) of vitrified ASCT2_C467R_ in absence of substrate. **c**, Angular distribution plot of particles included in the final C3-symmetrized 3D reconstruction. **d**, Image processing work flow. **e**, Final reconstructed map coloured by local resolution as estimated in Relion. **f**, FSC plot used for resolution estimation and model validation. The gold-standard FSC plot between two separately refined half-maps is shown in pink and indicates a final resolution of 4.1 Å. The FSC model validation curves for FSCsum, FSCwork and FSCfree, as described in material and methods, are shown in light pink, dark grey and light grey respectively. A thumbnail of the mask used for FSC calculation overlaid on the map is shown in the upper right corner. Dashed lines indicate the FSC thresholds used for FSC of 0.143 and for FSCsum of 0.5. **g**, Anisotropy estimation plot of the final map. The global FSC curve is represented in yellow. The directional FSCs along the x, y and z axis are displayed in blue, green and red, respectively.

**
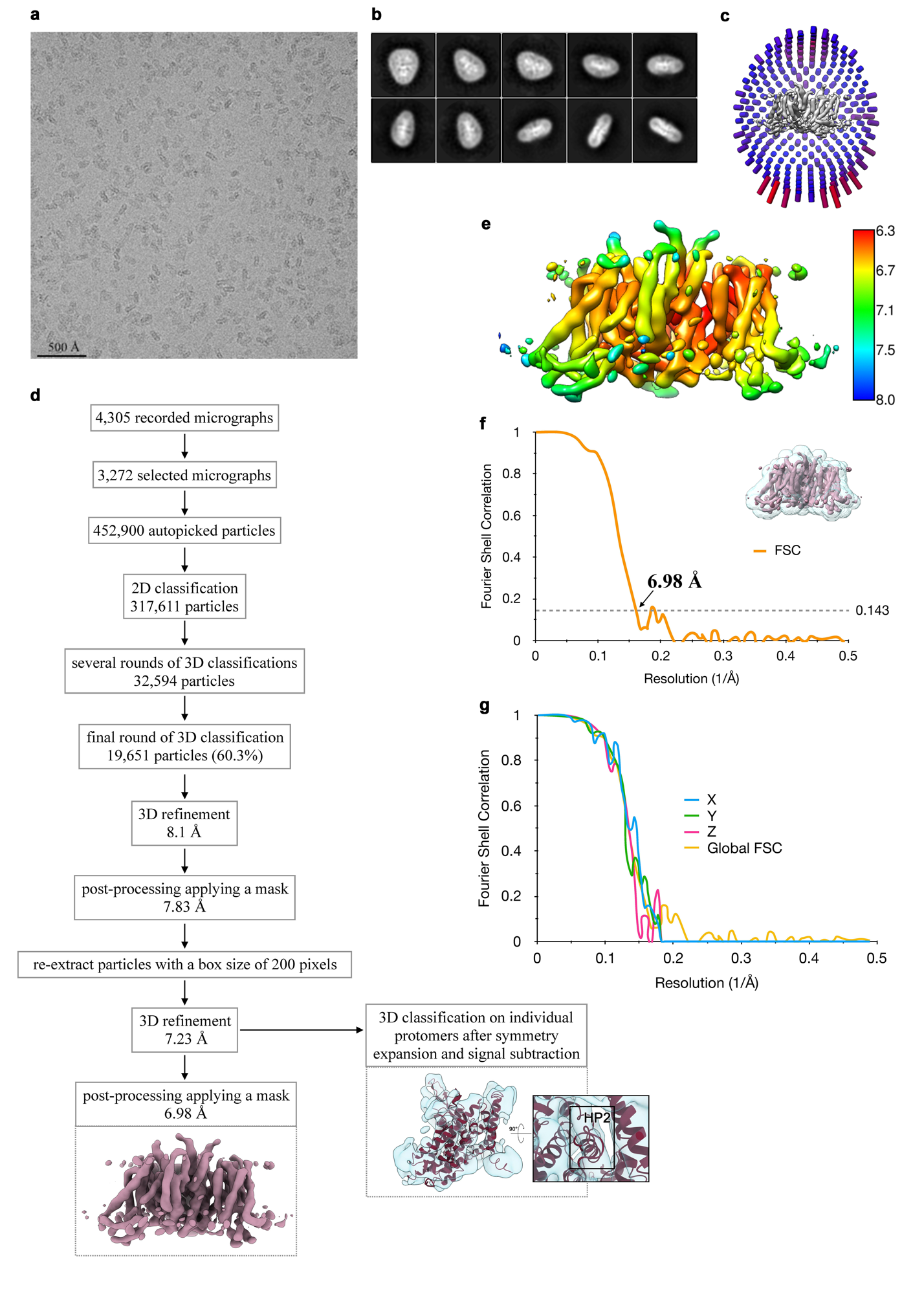
**

**Supplementary Figure 3: Cryo-EM reconstruction of substrate-free ASCT2_wt_**. Representative cryo-EM image (**a**) and 2D-class averages (**b**) of vitrified ASCT2_wt_ in absence of substrate. **c**, Angular distribution plot of particles included in the final C3-symmetrized 3D reconstruction. **d**, Image processing work flow. **e**, Final reconstructed map coloured by local resolution as estimated by Relion. **f**, FSC plot used for resolution estimation. The gold-standard FSC plot between two separately refined half-maps is shown in orange and indicates a final resolution of 7 Å. **g**, Anisotropy estimation plot of the final map. The global FSC curve is represented in yellow. The directional FSCs along the x, y and z axis are displayed in blue, green and red, respectively.

**
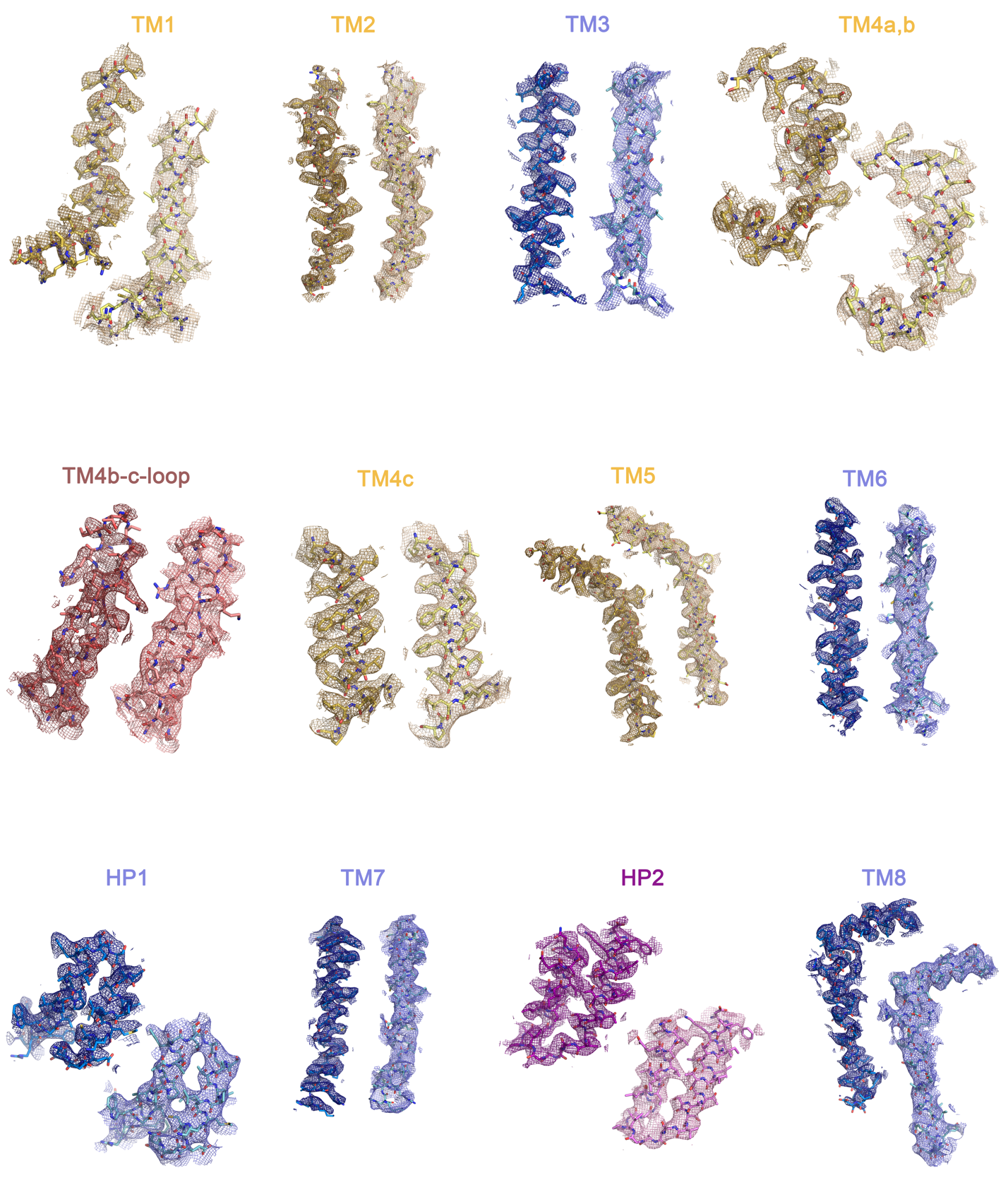
**

**Supplementary Figure 4: Cryo-EM densities**. Shown are selections of cryo-EM densities of ASCT2_C467R_ in presence of TBOA (left, dark colors) and substrate-free ASCT2_C467R_ (right, light colors), with the respective refined models superimposed. Models are shown as sticks and structural elements are labelled. Transmembrane helices (TM) of the transport domain are coloured in blue, of the scaffold domain in yellow, hairpin 2 (HP2) in purple and the loop between TM4b and TM4c is shown in red. Densities were sharpened with a b-factor of -204.52 Å^2^ (for ASCT2_C467R_- TBOA) and -211.195 Å^2^ (for substrate-free ASCT2_C467R_) and plotted at 3 σ, except for TM5, HP1, and HP2 of substrate-free ASCT2_C467R_, which were contoured at 4 σ.


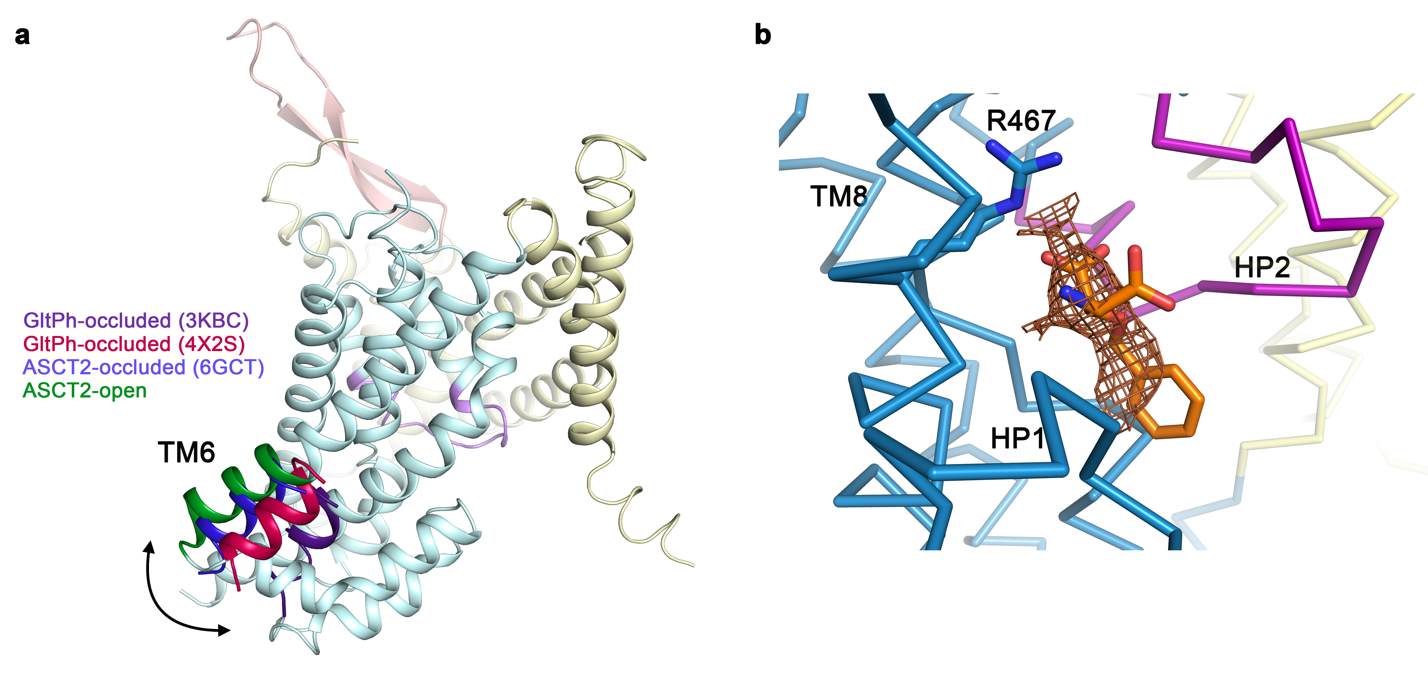


**Supplementary Figure 5: Structural properties of inward-open ASCT2_C467R_ in presence of TBOA. a**, Structural comparison of Glt_Ph_ and ASCT2 inward-facing states, when superimposed on the scaffold domain TM2, 4, 5. The protomer of the inward-open ASCT2_C467R_ is shown as ribbon and color-coded (transport domain in light blue, scaffold domain in light yellow, antenna in light red and the HP2 loop in light purple). Only the C-terminal tip of TM6 is shown for the other inward-facing SLC1A structures and colored as indicated. The tip of TM6 serves as an indication for the movement of the transport domain, revealing that it is further tilted towards the cytoplasm in the inward-open state. **b,** Weak non-protein cryo-EM density that likely represents a low-occupancy of TBOA in the binding site of ASCT2_C467R_ is shown as orange mesh contoured at 2 σ.


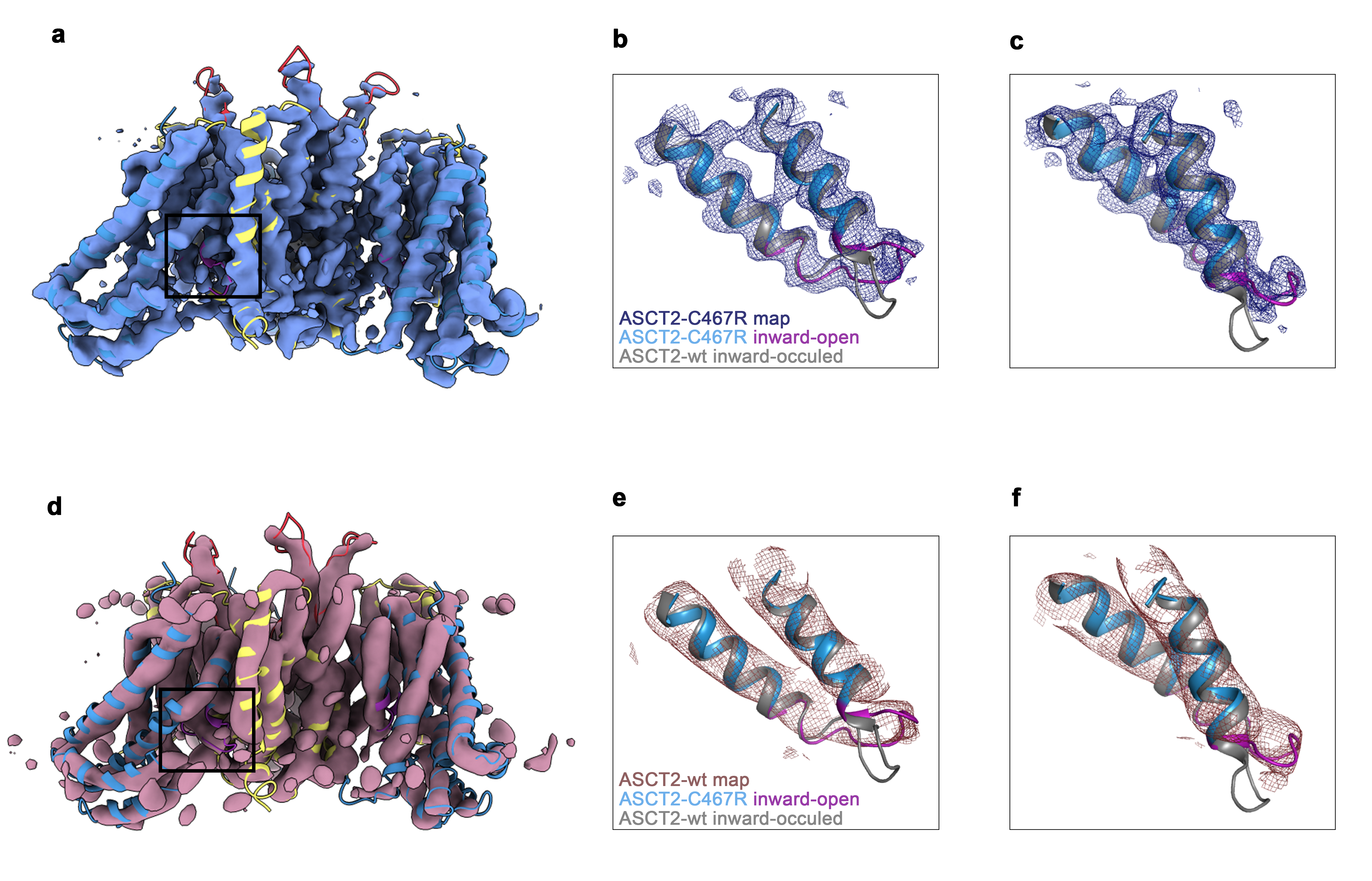


**Supplementary Figure 6: Cryo-EM maps of ASCT2_C467R_ and ASCT2_wt_ in absence of substrate show HP2 in the inward-open conformation. a**, Superposition of the cryo-EM map of substrate-free ASCT2_C467R_ at 4.1 Å resolution and its respective model (color-coded: transport domain in blue, scaffold domain in yellow, antennae in red and the HP2 loop in purple). The HP2 position in the transport domain is highlighted by a box. **b, c**, Superposition of the cryo-EM density for HP2 in the substrate-free ASCT2_C467R_ map (shown as mesh at 4.5 σ) with its respective model in an inward-open conformation (blue ribbon with the HP2 loop in purple) and the substrate-bound inward-occluded ASCT2_wt_ model (dark grey, PDB-ID: 6GCT). **d**, Superposition of the cryo-EM map of substrate-free ASCT2_wt_ at 7 Å resolution and its respective model (color-coded as in a). The HP2 position in the transport domain is highlighted by a box. **e, f**, Superposition of the cryo-EM density for HP2 in the substrate-free ASCT2_wt_ map (shown as mesh at 4 σ), with its respective model in an inward-open conformation (blue ribbon with the HP2 loop in purple) and the substrate-bound inward-occluded ASCT2_wt_ model (dark grey, PDB-ID: 6GCT).

**
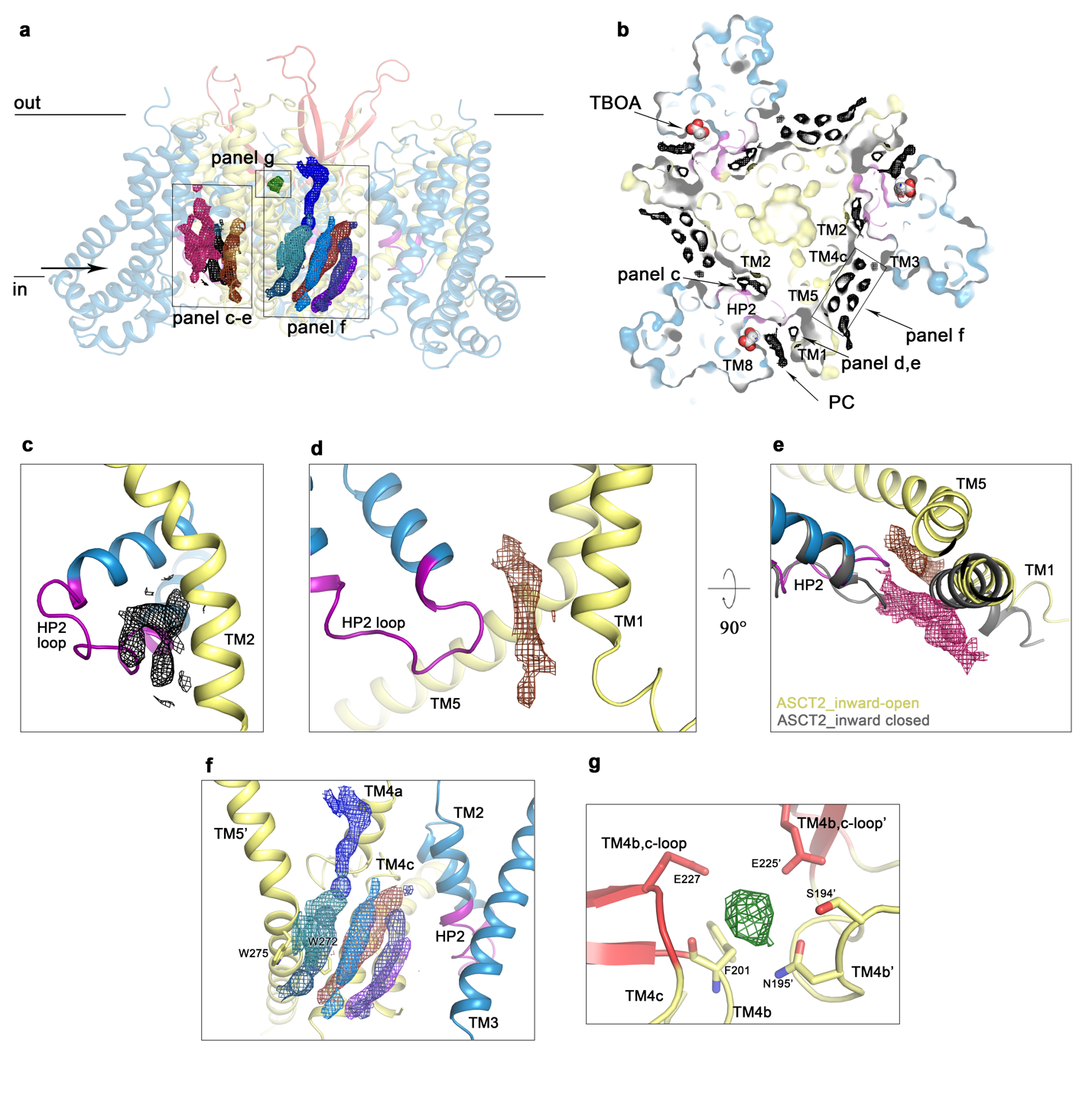
**

**Supplementary Figure 7: Non-protein densities in ASCT2_C467R_. a,** Ribbon representation of the ASCT2_C467R_-TBOA with the scaffold domains coloured in pale yellow, the transport domains in pale blue, the extracellular antennae in pale red and the HP2 loop in pale purple. Non-protein densities are shown as coloured mesh at 3 σ (panels c-e) and 4 σ (panel f, g). They are mainly located on the inner membrane leaflet, and most likely represent lipid or detergent molecules (panels c-f). **b**, Slice through view of ASCT2_C467R_ (level is indicated by an arrow in panel a, with the putative lipid densities surrounding the scaffold domain shown in black and TBOA as balls. **c**, Density located at the interface of HP2 and TM2 of the scaffold domain. It is located roughly at the same location as the putatively bound cholesterols found in the inward-occluded ASCT2_wt_ structure^2^. **d**, **e**, Density located between TM1 of the scaffold domain, HP2 of the transport domain and the phosphatidylcholine (PC) located at the tip of both hairpins. It potentially represents a lipid molecule and might cause TM1 to relocate when compared with the inward-occluded ASCT2_wt_ structure. **f**, Array of non-protein densities, located close to the scaffold domains. The most right, dark purple density is located at a similar region as the allosteric inhibitor UCPH_101_ found in the EAAT1 structure^19^. **g**, Non-protein density, located inside the extracellular bowl sandwiched between the N- and C-terminus of two adjacent antennae (TM4b,c-loop). Panels c-e represent zoomed views of panel a.
