# Supplementary material for: A one-gate elevator mechanism for the human neutral amino acid transporter ASCT2
Source: Nat Commun. 2019 Jul 31;10:3427. doi: 10.1038/s41467-019-11363-x (PMC6668440; doi:10.1038/s41467-019-11363-x)
Supplement: Supplementary file 3 — Description of Additional Supplementary Files [file 41467_2019_11363_MOESM3_ESM.docx]

|  |  |  |
| --- | --- | --- |
|  |  |  |

**Title: Supplementary Movie 1:** **One-gate elevator mechanism.
Description:** Shown is a morph between the outward-open EAAT1 (PDB-ID 5MJU), the outward-occluded EAAT1 (PDB-ID 5LLU), the inward-occluded ASCT2 (PDB-ID 6GCT) and the inward-open ASCT2 when superimposed on the scaffold domain TM2, 4, 5, elucidating the one-gate elevator mechanism (Fig. 5). The scaffold domains are coloured in yellow, the transport domains in blue and HP2 in purple. Throughout the transport cycle the transport domain behaves as a rigid body, except for the movement of the HP2 loop. Note that the observed movement of TM1 from the inward-occluded to the inward-open state is not the result of a direct interaction with HP2, but might be linked to putative lipid interaction found between TM1 and HP2 (Supplementary Figure 7b,e).
