# Supplementary material for: A one-gate elevator mechanism for the human neutral amino acid transporter ASCT2
Source: Nat Commun. 2019 Jul 31;10:3427. doi: 10.1038/s41467-019-11363-x (PMC6668440; doi:10.1038/s41467-019-11363-x)
Supplement: Supplementary file 4 — Reporting Summary [file 41467_2019_11363_MOESM4_ESM.pdf]

nature research

Corresponding author(s): Dirk J. Slotboom, Cristina Paulino

Last updated by author(s): Jun 4, 2019

# Reporting Summary

Nature Research wishes to improve the reproducibility of the work that we publish. This form provides structure for consistency and transparency in reporting. For further information on Nature Research policies, see Authors & Referees and the Editorial Policy Checklist.

## Statistics

For all statistical analyses, confirm that the following items are present in the figure legend, table legend, main text, or Methods section.

| n/a | Confirmed | |
|---|---|---|
| ☐ | ☒ | The exact sample size (*n*) for each experimental group/condition, given as a discrete number and unit of measurement |
| ☐ | ☒ | A statement on whether measurements were taken from distinct samples or whether the same sample was measured repeatedly |
| ☒ | ☐ | The statistical test(s) used AND whether they are one- or two-sided<br>*Only common tests should be described solely by name; describe more complex techniques in the Methods section.* |
| ☒ | ☐ | A description of all covariates tested |
| ☒ | ☐ | A description of any assumptions or corrections, such as tests of normality and adjustment for multiple comparisons |
| ☒ | ☐ | A full description of the statistical parameters including central tendency (e.g. means) or other basic estimates (e.g. regression coefficient) AND variation (e.g. standard deviation) or associated estimates of uncertainty (e.g. confidence intervals) |
| ☒ | ☐ | For null hypothesis testing, the test statistic (e.g. *F*, *t*, *r*) with confidence intervals, effect sizes, degrees of freedom and *P* value noted<br>*Give P values as exact values whenever suitable.* |
| ☒ | ☐ | For Bayesian analysis, information on the choice of priors and Markov chain Monte Carlo settings |
| ☒ | ☐ | For hierarchical and complex designs, identification of the appropriate level for tests and full reporting of outcomes |
| ☒ | ☐ | Estimates of effect sizes (e.g. Cohen's *d*, Pearson's *r*), indicating how they were calculated |

*Our web collection on statistics for biologists contains articles on many of the points above.*

## Software and code

Policy information about availability of computer code

| | |
|---|---|
| Data collection | The commercial available software EPU from Thermo Fisher Scientific was used for automated cryo-EM data collection. Data was processed on the fly using the open source software FOCUS. |
| Data analysis | Cryo-EM data was analysed using the software MotionCor2_1.1.0, ctffind4.1.8, RELION-3.0-beta-2. Model was built using the software Coot, Phenix. Visualization was performed with the software Pymol, Chimera, ChimeraX. SBGrid was used to install all mentions software. |

For manuscripts utilizing custom algorithms or software that are central to the research but not yet described in published literature, software must be made available to editors/reviewers. We strongly encourage code deposition in a community repository (e.g. GitHub). See the Nature Research guidelines for submitting code & software for further information.

## Data

Policy information about availability of data

All manuscripts must include a data availability statement. This statement should provide the following information, where applicable:

- Accession codes, unique identifiers, or web links for publicly available datasets
- A list of figures that have associated raw data
- A description of any restrictions on data availability

The three-dimensional cryo-EM density maps of the inward-open ASCT2-C467R in presence of TBOA, apo ASCT2-C467R and apo ASCT2wt have been deposited in the Electron Microscopy Data Bank under accession numbers EMD-10016, EMD-10018 and EMD-10017, respectively. The deposition includes the cryo-EM maps, both half-maps, the unmasked and unsharpened refined maps and the mask used for final FSC calculation. Coordinates of two models have been deposited in the Protein Data Bank. The accession numbers for ASCT2C467R in presence of TBOA and apo ASCT2C467R are 6RVX and 6RVY, respectively.

# Field-specific reporting

Please select the one below that is the best fit for your research. If you are not sure, read the appropriate sections before making your selection.

☒ Life sciences ☐ Behavioural & social sciences ☐ Ecological, evolutionary & environmental sciences

For a reference copy of the document with all sections, see nature.com/documents/nr-reporting-summary-flat.pdf

# Life sciences study design

All studies must disclose on these points even when the disclosure is negative.

| | |
|---|---|
| Sample size | Uptake assays represent at least three biological replicates |
| Data exclusions | No data was excluded |
| Replication | All attempts at replication were successful and reproducible |
| Randomization | Samples were not allocated into groups, thus randomization is not relevant for this study |
| Blinding | Blinding is not relevant for this study since there was no group allocation |

# Reporting for specific materials, systems and methods

We require information from authors about some types of materials, experimental systems and methods used in many studies. Here, indicate whether each material, system or method listed is relevant to your study. If you are not sure if a list item applies to your research, read the appropriate section before selecting a response.

## Materials & experimental systems

| n/a | Involved in the study |
|---|---|
| ☒ | ☐ Antibodies |
| ☒ | ☐ Eukaryotic cell lines |
| ☒ | ☐ Palaeontology |
| ☒ | ☐ Animals and other organisms |
| ☒ | ☐ Human research participants |
| ☒ | ☐ Clinical data |

## Methods

| n/a | Involved in the study |
|---|---|
| ☒ | ☐ ChIP-seq |
| ☒ | ☐ Flow cytometry |
| ☒ | ☐ MRI-based neuroimaging |
